# Untargeted NMR-Based Methodology in the Study of Fruit Metabolites

**DOI:** 10.3390/molecules20034088

**Published:** 2015-03-04

**Authors:** Anatoly Petrovich Sobolev, Luisa Mannina, Noemi Proietti, Simone Carradori, Maria Daglia, Anna Maria Giusti, Riccarda Antiochia, Donatella Capitani

**Affiliations:** 1 Magnetic Resonance Laboratory “Annalaura Segre”, Institute of Chemical Methodologies, National Research Council (CNR), I-00015 Monterotondo (RM), Italy; E-Mails: anatoly.sobolev@cnr.it (A.P.S.); noemi.proietti@cnr.it (N.P.); donatella.capitani@cnr.it (D.C.); 2 Department of Drug Chemistry and Technologies, Sapienza University of Rome, Piazzale Aldo Moro 5, I-00185 Rome, Italy; E-Mail: riccarda.antiochia@uniroma1.it; 3 Department of Pharmacy, “G. D’Annunzio” University of Chieti-Pescara, Via dei Vestini 31, 66100 Chieti Scalo (CH), Italy; E-Mail: simone.carradori@unich.it; 4 Department of Drug Sciences, Medicinal Chemistry and Pharmaceutical Technology Section, Pavia University, Via Taramelli 12, 27100 Pavia, Italy; E-Mail: maria.daglia@unipv.it; 5 Department of Experimental Medicine, Sapienza University of Rome, Piazzale Aldo Moro 5, I-00185 Rome, Italy; E-Mail: annamaria.giusti@uniroma1.it

**Keywords:** NMR, fruits, metabolomics, primary metabolites, secondary metabolites

## Abstract

In this review, fundamental aspects of the untargeted NMR-based methodology applied to fruit characterization are described. The strategy to perform the structure elucidation of fruit metabolites is discussed with some examples of spectral assignments by 2D experiments. Primary ubiquitous metabolites as well as secondary species-specific metabolites, identified in different fruits using an untargeted ^1^H-NMR approach, are summarized in a comprehensive way. Crucial aspects regarding the quantitative elaboration of spectral data are also discussed. The usefulness of the NMR-based metabolic profiling was highlighted using some results regarding quality, adulteration, varieties and geographical origin of fruits and fruit-derived products such as juices.

## 1. Introduction

All fruits have a specific chemical profile mostly characterized by endogenous components (produced by the plant and by environmental microorganisms) that are strongly connected to their nutritional value, aroma, taste and health-promoting effects. These fruit components can be ubiquitous primary metabolites (such as amino acids, sugars, organic acids) involved in the basic functions of the living cells or secondary metabolites that are usually fruit-type specific. Taken together these metabolites can be used as potential markers for quality, origin and authenticity of fruit and fruit-derived foods. 

Taking into account the great variety of fruit-derived foodstuffs, numerous chemical and physico-chemical methods of analysis have been proposed to control their sensorial and nutritional properties, quality and authenticity [1]. Analytical methodologies, called targeted analyses, focus on a specific class of compounds or single compounds significant for assessing foodstuff quality, nutritional/sensorial properties, and biological characteristics. In the case of targeted analyses, selective extractions and/or separations are usually performed before the analysis to isolate and concentrate the selected metabolites and to avoid interference from other compounds. The targeted approach provides reliable and sensitive identification and precise and accurate quantification of specific compounds. In literature, many analytical methodologies such as chromatography, spectrophotometry, and spectroscopic techniques have been applied to determine specific fruits metabolites. Typical fruit target compounds are phenolics, sugars, amino acids and organic acids. 

Other analytical methodologies, called untargeted analyses, are not focused on specific target compounds and try to give a picture as complete as possible of the chemical composition of the investigated sample. In the case of untargeted analyses, metabolite extraction from fruits is carried out choosing the solvent or the mixture of solvent able to extract the highest number of metabolites. 

Metabolomics, as a non-biased identification and quantification of all metabolites present in a given matrix, uses untargeted analytical methodologies [2]. In metabolomics a wide range of different compounds with different chemical natures, solubility and concentration has to be analyzed. Generally, fruit metabolomics is extremely complex due to the enormous diversity of metabolite chemical structures present in plants, especially among the secondary metabolites which are specific for every species. In the last few years, metabolomic studies have experienced a notable increase in interest, producing many important results from an analytical point of view [3]. Among the untargeted analytical methodologies used to analyze fruits, NMR-based metabolomics has gained an important role because it can simultaneously bring “high-throughput” spectroscopic/structural information on a wide range of metabolites with a high analytical precision [4] avoiding biases against certain classes of compounds. The ^1^H-NMR spectra of fruits extracts are inevitably crowded because of the presence of many compounds. However, NMR is also especially helpful for the identification of secondary metabolites in fruit extracts by means of 1D and 2D experiments, standard additions and by comparison with literature databases. 

In this review, some important aspects (spectral assignments, quantitative analysis and statistical elaboration) of the use of NMR methodology for fruit extract characterization are reported together with an overview of the current literature data regarding the metabolites identified by the untargeted NMR approach. The usefulness of the metabolite profiling in variety discrimination and in the investigation of fruit development and ripening, quality and geographical origin is evidenced. 

## 2. Metabolic Profile: Spectral Assignment, Quantitative Analysis and Statistical Elaboration 

### 2.1. Spectral Assignment

**Figure 1:**
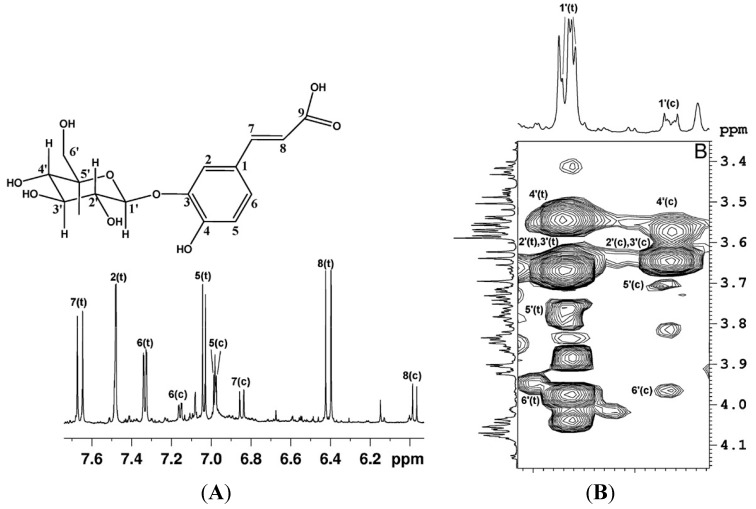
(**A**) ^1^H expanded spectral region and assignment of aromatic and vinyl protons of O^3^-β-glucopyranosyl-*trans*-caffeic acid and O^3^-β-glucopyranosyl-*cis*-caffeic acid (c = *cis*; t = *trans*). (**B**) Expansion of ^1^H-^1^H TOCSY map, the proton spin system of the glucopyranosyl moiety of O^3^-β-glucopyranosylcaffeic acid is shown (reproduced with permission from [6]).

Fruit composition is extremely complex due to the enormous diversity of metabolite chemical structures present in fruits, especially regarding the secondary metabolites specific for each botanical species. As a consequence, highly crowded ^1^H-NMR spectra of fruit extracts are usually obtained owing to the presence of hundreds of multiplet resonances. In order to simplify the spectra and to disentangle overlapped signals, the spectral assignment strategies inevitably include two-dimensional NMR experiments such as homonuclear correlations like ^1^H-^1^H COSY and/or ^1^H-^1^H TOCSY, or heteronuclear correlations like ^1^H-^13^C HSQC and ^1^H-^13^C HMBC [5]. Among homonuclear correlation experiments, ^1^H-^1^H TOCSY is particularly feasible for mixture analysis allowing the assignment of all hydrogen signals within a spin system that forms an unbroken chain of couplings, thereby entire molecules or molecular fragments can be assigned, see Figure 1.

Heteronuclear ^1^H-^13^C correlation experiments have the advantage of spreading the metabolite signals in the ^13^C dimension over a broader range (0–200 ppm) respect to that of ^1^H dimension (0–10 ppm) and to observe indirectly ^13^C spectrum with higher sensitivity through the use of inverse detection. Apart from various 2D-experiments, diffusion ordered NMR spectroscopy (DOSY) [7] offers an additional support for the structure elucidation of complex mixtures giving information regarding the self-diffusion coefficients of the metabolites present in the sample. DOSY is a particularly convenient way of displaying the molecular self-diffusion information in a bidimensional array, with the NMR spectrum in one dimension and the self-diffusion coefficient in the other one. Therefore, different components in a fruit extract can be distinguished by their self-diffusion coefficients as reported for fruit juices by Gil *et al.* [8]. The detailed description of NMR spectral assignment methods applied to complex mixtures can be found elsewhere [9,10].

Using the NMR approach it has been possible to identify primary as well as secondary metabolites of different fruits such as grape, orange, apple juice, mandarin orange, kiwifruits, mango, black raspberry, melon, watermelon, blueberry and peaches, see Table 1. The presence of a metabolite in a given foodstuff is indicated by the letter X. In this type of NMR based analyses, the extraction procedure is probably the most critical step aimed to the quantitative transfer of the metabolites from the solid matrix into the solution. Note that the major part of metabolites has been extracted and identified in aqueous solutions. In the case of phenolic compounds poorly soluble in water, the mixture of water and methanol is usually used for extraction and subsequent NMR analysis. In some specific cases, in order to obtain high resolution NMR spectra, methanol with trifluoroacetic acid has been used for fruit extraction and then deuterated methanol as NMR solvent [11].

**Figure 2:**
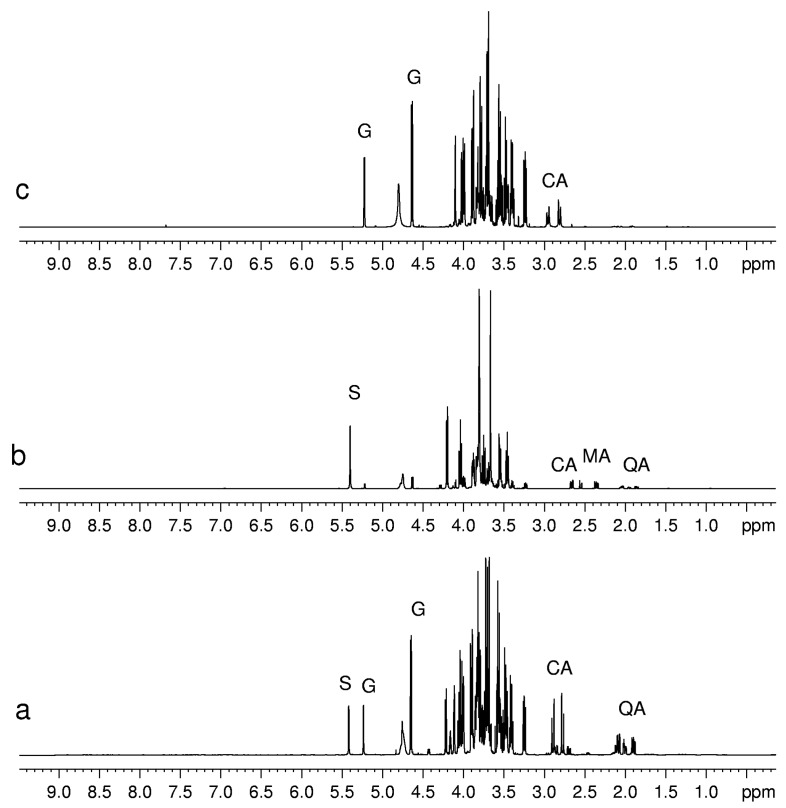
^1^H-NMR spectra of aqueous extracts of (**a**) kiwifruits; (**b**) peach fruits; (**c**) blueberry fruits. Abbreviations: S, sucrose; G, glucose; CA, citric acid; MA, malic acid; QA, quinic acid.

Each fruit shows a characteristic set of primary ubiquitous metabolites (such as amino acids, sugars, and organic acids) involved in the basic functions of living cells. Anyway, the amount of primary metabolites varies in different fruits. These differences can be directly observed in ^1^H-NMR spectra (see as an example Figure 2 in which the ^1^H-NMR spectra of kiwifruits, blueberries and peaches are compared). 

**Figure 3:**
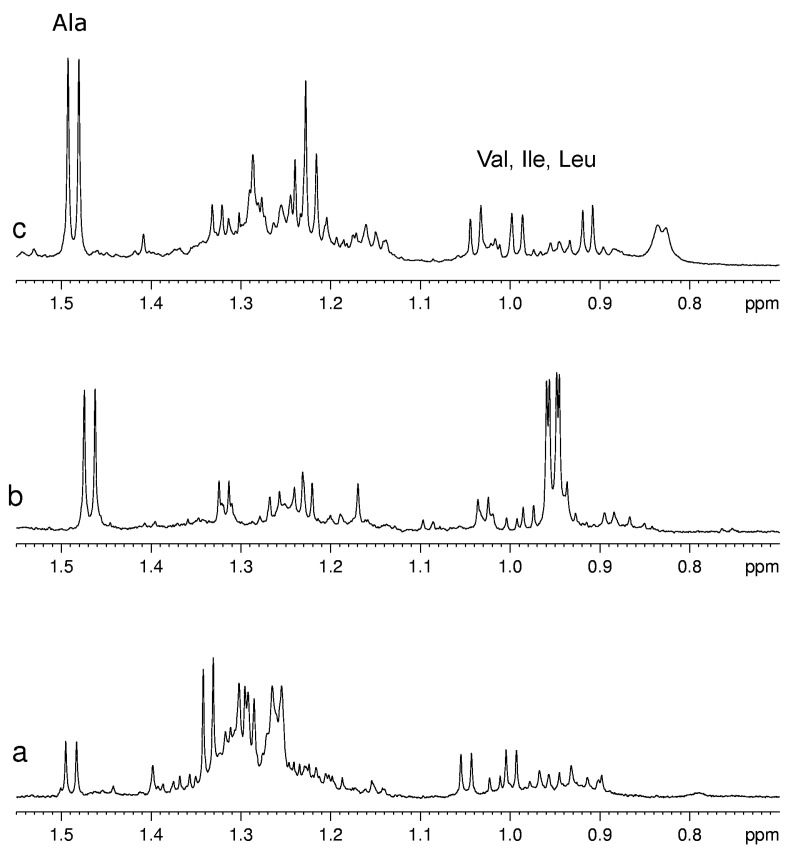
A selected region of ^1^H-NMR spectra of aqueous extracts of (**a**) kiwifruits; (**b**) peach fruits; (**c**) blueberry fruits.

Usually for the assignment of a specific metabolite in the complex spectra of fruit extracts at least one signal of this metabolite has to be resolved from other signals in the spectrum. Unfortunately, not all metabolites have this “diagnostic” signal. For example, pyruvate has only one signal from its CH_3_ group in the range 2.35–2.40 ppm that usually is overlapped with the multiplet signals of other almost ubiquitous metabolites such as glutamate and glutamine. As a consequence, only a relatively high concentration of pyruvate is detectable, therefore its detection limit in mixture is substantially higher than that for a single compound. On the other hand, the metabolites with characteristic signals not overlapped with other components are well detectable also when they are present in low concentration as for example Val, Leu, Ile and Ala (see Figure 3).

**Table 1:** Metabolites identified in ^1^H-NMR spectra of fruit extracts.

Metabolite	Grape ^a^	Orange Juice ^b^	Apple Juice ^c^	Mandarin Orange ^d^	Kiwifruit ^e^	Mango ^f^	Black Raspberry ^g^	Melon ^h^	Watermelon ^i^	Blueberry ^j^	Peach ^k^	
*Acids*												
**Acetic**	**X**		**X**				**X**	**X**				
**Ascorbic**	**X**	****	****	**X**	**X**		**X**					
**Citramalic**			**X**									
**Citric**	**X**	**X**	**X**	**X**	**X**	**X**	**X**	**X**	**X**	**X**	**X**	
**3,4-Dihydroxybenzoic**							**X**					
**Formic**	**X**		**X**	**X**		**X**	****	****	****	****	****	
**Fumaric**	**X**	****	****	****	****	**X**	**X**	**X**	**X**	****	**X**	
**Glutaric**	****	****	****	****	****	****	**X**					
**3-Hydroxybutyric**						**X**	**X**					
**Lactic**	**X**		**X**		**X**	**X**	**X**	****	****	****	****	
**Maleic**	****	****	****	****	****	****	**X**	****	****	****	****	
**Malic**	**X**	**X**	**X**	****	**X**	**X**	****	**X**	**X**	**X**	**X**	
**Methyl-malonic**						**X**	****	****	****	****	****	
**Pyruvic**	****	****	****	****	****	****	**X**	****	****	****	****	
**Quinic**	****	**X**	**X**	****	**X**	**X**	****	****	****	**X**	**X**	
**Shikimic**					**X**	**X**				**X**	**X**	
**Succinic**	**X**	**X**	**X**	**X**	****	**X**	**X**	****	****	****	**X**	
**Tartaric**	**X**	****	**X**	****	****	**X**	****	****	****	****	****	
*Sugars and sugar alcohols*												
**β-Arabinose**	****	****	****	****	****	**X**	****	****	****	****	****	
**α-d-Fructofuranose**	**X**	****	**X**	**X**	**X**	**X**	**X**	**X**	**X**	**X**	**X**	
**β-d-Fructofuranose**	****	**X**	****	****	**X**	****	****	****	****	**X**	**X**	
**β-d-Fructopyranose**	****	****	****	****	**X**	****	****	****	****	**X**	**X**	
**Fructose-6P**	****	****	****	****	**X**	****	****	****	****	****	**X**	
**Fucose**	****	****	****	****	****	**X**	****	****	****	****	**X**	
**α-Galactose**	****	****	**X**	****	**X**	****	****	**X**	****	****	****	
**β-Galactose**	****	****	**X**	****	**X**	**X**	****	****	****	****	****	
**α-Glucose**	**X**	**X**	**X**	**X**	**X**	**X**	**X**	**X**	**X**	**X**	**X**	
**β-Glucose**	**X**	**X**	**X**	**X**	**X**	**X**	**X**	**X**	**X**	**X**	**X**	
**α-Glucose-6P**	****	****	****	****	**X**	****	****	****	****	****	**X**	
**β-Glucose-6P**	****	****	****	****	**X**	****	****	****	****	****	**X**	
**Limonin glucoside**	****	****	****	**X**	****	****	****	****	****	****	****	
**α-Mannose**	****	****	****	****	**X**	****	****	****	****	****	****	
**β-Mannose**	****	****	****	****	**X**	****	****	****	****	****	****	
***Myo*-inositol**	****	****	****	**X**	**X**	****	****	**X**	****	**X**	**X**	
**Raffinose**	****	****	****	****	**X**	****	****	****	****	****	****	
**α-Rhamnose**	****	****	****	****	****	****	****	****	****	****	**X**	
**β-Rhamnose**	****	****	****	****	****	**X**	****	****	****	****	**X**	
**Stachyose**	****	****	****	****	****	****	****	**X**				
**Sucrose**	**X**	**X**	**X**	**X**	**X**	**X**	**X**	**X**	**X**	**X**	**X**	
**d-Trehalose**								**X**				
**α-Xylose**			**X**		**X**						**X**	
**β-Xylose**			**X**		**X**						**X**	
*Amino acids, peptides and derivatives*												
**Alanine**	**X**	**X**	**X**	**X**	**X**	**X**	**X**	**X**		**X**	**X**	
**γ-Amino-butyrate**	**X**	**X**	**X**	**X**	**X**	**X**	****	**X**	**X**	**X**	****	
**Arginine**	**X**	**X**	****	**X**	**X**	**X**	**X**	****	****	**X**	****	
**Asparagine**	****	**X**	**X**	**X**	**X**	**X**	**X**	**X**	****	**X**	**X**	
**Aspartate**	****	**X**	**X**	**X**	**X**	**X**	**X**	**X**	****	****	****	
**Citrulline**							**X**		**X**	****	****	
**Dimethyl-proline**	****	**X**	****	****	****	****	****	****	****	****	****	
**Glutamate**	**X**	**X**	**X**	****	**X**	**X**	**X**	**X**	****	**X**	****	
**Glutamine**	**X**	**X**	****	****	**X**	**X**	**X**	**X**	**X**	**X**	****	
**Glycine**	****	****	****	****	****	****	**X**	****	****	****	****	
**Histidine**	****	**X**	****	**X**	**X**	****	****	****	****	****	****	
**Isoleucine**	**X**	****	**X**	**X**	**X**	**X**	**X**	**X**	**X**	****	**X**	
**Leucine**	**X**	****	**X**	**X**	**X**	**X**	**X**	****	****	**X**	****	
**Lysine**	****	****	****	****	**X**	**X**						
**Methionine**	**X**											
**Ornithine**		**X**				**X**						
**Phenylalanine**		**X**		**X**	**X**	**X**	**X**	**X**			**X**	
**Proline**	**X**	**X**	****	**X**	****	****	**X**	****	****	****	****	
**Proline-betaine**	****	****	****	**X**	****	****	****	****	****	****	****	
**Pyroglutamate**	****	****	****	****	****	****	****	**X**				
**Threonine**	**X**	**X**		**X**	**X**	**X**		**X**		**X**	**X**	
**Tryptophane**					**X**			**X**				
**Tyrosine**		**X**					**X**	**X**				
**Valine**	**X**	**X**	**X**	**X**	**X**	**X**	**X**	**X**	**X**	**X**	**X**	
*Alcohols, polyols, amines, aldehydes, ketones, esters*	****	****	****	****	****	****	****	****	****	****	****	
**Acetaldehyde hydrate**	****	****	****	****	****	**X**	****	****	****	****	****	
**Benzoic acid β** **-d-glucopyranosyl ester**	****	****	****	****	****	****	**X**	****	****	****	****	
**2,3-Butanediol**	**X**											
**Choline**	**X**			**X**	**X**	**X**		**X**		**X**	**X**	
**Ethanol**		**X**	**X**	**X**		**X**		**X**				
**Galactinol**								**X**				
**α-Glycerophosphoryl choline**											**X**	
**Histamine**							**X**					
**Methanol**				**X**		**X**						
**Methyl-amine**						**X**						
**Propanol**			**X**									
**L-Rhamnitol**			**X**									
**Salicylic acid β-d-glucopyranosyl ester**							**X**					
*Nucleic acid derivatives*											
**Adenine**						**X**					
**Adenosine**				**X**							
**Adenosine-MP**								**X**			
**ATP**					**X**						**X**
**Uridine**					**X**						**X**
*Aromatic Compounds*											
**Caffeic acid**							**X**				
***cis*-Caftaric acid**	**X**										
***trans*-Caftaric acid**	**X**										
**(+)-Catechin**	**X**										**X**
**Chlorogenic acid**	****	****	**X**	****	****	****	****	****	****	**X**	**X**
**Cinnamic acid**	****	****	****	****	****	****	**X**	****	****	****	****
***p*-Coumaric acid**	****	****	**X**	****	****	****	**X**	****	****	****	****
***p*-Coumaryl glucoside**	****	****	****	****	****	****	**X**	****	****	****	****
***cis-p*-Coutaric acid**	**X**	****	****	****	****	****	****	****	****	****	****
***trans-p*-Coutaric acid**	**X**	****	****	****	****	****	****	****	****	****	****
**Cyanidin-3-glucoside**	****	****	****	****	****	****	**X**	****	****	****	****
**Cyanidin-3-rutinoside**	****	****	****	****	****	****	**X**	****	****	****	****
**Cyanidin-3-sambubioside**	****	****	****	****	****	****	**X**	****	****	****	****
**Cyanidin-3-xylosylrutinoside**	****	****	****	****	****	****	**X**	****	****	****	****
**Delphinidin-3-galactoside**	****	****	****	****	****	****	****	****	****	**X**	****
**Delphinidin-3-glucoside**	****	****	****	****	****	****	****	****	****	**X**	****
**Dihydrokaempferol glucoside**	****	****	****	****	****	****	**X**	****	****	****	****
**Dihydrosinapic acid**							**X**				
**Ellagic acid**							**X**				
**(−)-Epicatechin**	**X**		**X**		**X**		**X**				
***trans*-Fertaric acid**	**X**										
**Ferulic acid**	****	****	****	****	****	****	**X**	****	****	****	****
**Gallic acid**	**X**					**X**	**X**				
**GLUcCA**	****	****	****	****	**X**	****	****	****	****	****	****	
**GLUtCA**	****	****	****	****	**X**	****	****	****	****	****	****	
**Malvidin-3-galactoside**	****	****	****	****	****	****	****	****	****	**X**	****	
**Malvidin-3-glucoside**	****	****	****	****	****	****	****	****	****	**X**	****	
**Methyl ellagic acid acetylpentose**	****	****	****	****	****	****	**X**	****	****	****	****	
**Myricetin**	**X**	****	****	****	****	****	****	****	****	****	****	
**Myricetin glucoside**	****	****	****	****	****	****	**X**	****	****	****	****	
**Narirutin**	****	**X**	****	****	****	****	****	****	****	****	****	
**Neochlorogenic acid**	****	****	****	****	**X**	****	****	****	****	****	**X**	
**Niacin**	****	****	****	****	****	**X**	****	****	****	****	****	
**Petunidin-3-glucoside**	****	****	****	****	****	****	****	****	****	**X**	****	
**Phloretin**	****	****	**X**	****	****	****	****	****	****	****	****	
**Phloridzin**	****	****	**X**	****	****	****	****	****	****	****	****	
***trans*-Piceid**	****	****	****	****	****	****	**X**	****	****	****	****	
**Protocatechuic acid**	****	****	****	****	****	****	**X**	****	****	****	****	
**Quercetin-3-*O*-glucoside**	**X**	****	****	****	****	****	**X**	****	****	****	****	
**Quercetin-3-*O*-rhamnoside**	****	****	****	****	**X**	****	****	****	****	**X**	****	
**Quercetin-3-*O*-rutinoside**	****	****	****	****	****	****	**X**	****	****	****	****	
**Synephrine**	****	****	****	**X**	****	****	****	****	****	****	****	
**Syringic acid**	**X**											
**Trigonelline**	**X**	****	****	**X**	****	****	****	**X**	****	****	**X**	
**Vanillic acid**	**X**						**X**					
*Fatty acids, lipids*												
**Fatty acids**							**X**					
**α-Linolenic A.**	**X**	****	****	****	****	****	****	****	****	****	****	
*Others*												
**β-Carotene**						**X**						

^a^ [12]; ^b^ [13]; ^c^ [14,15]; ^d^ [16]; ^e^ [6,17]; ^f^ [18]; ^g^ [11,19,20]; ^h^ [21]; ^i^ [22]; ^j^ [23]; ^k^ [24]. Abbreviations: ATP, adenosine-triphosphate; GLUtCA, O^3^-β-d-glucopyranosyl-*trans*-caffeic acid; GLUcCA, O^3^-β-d-glucopyranosyl-*cis*-caffeic acid.

**Figure 4:**
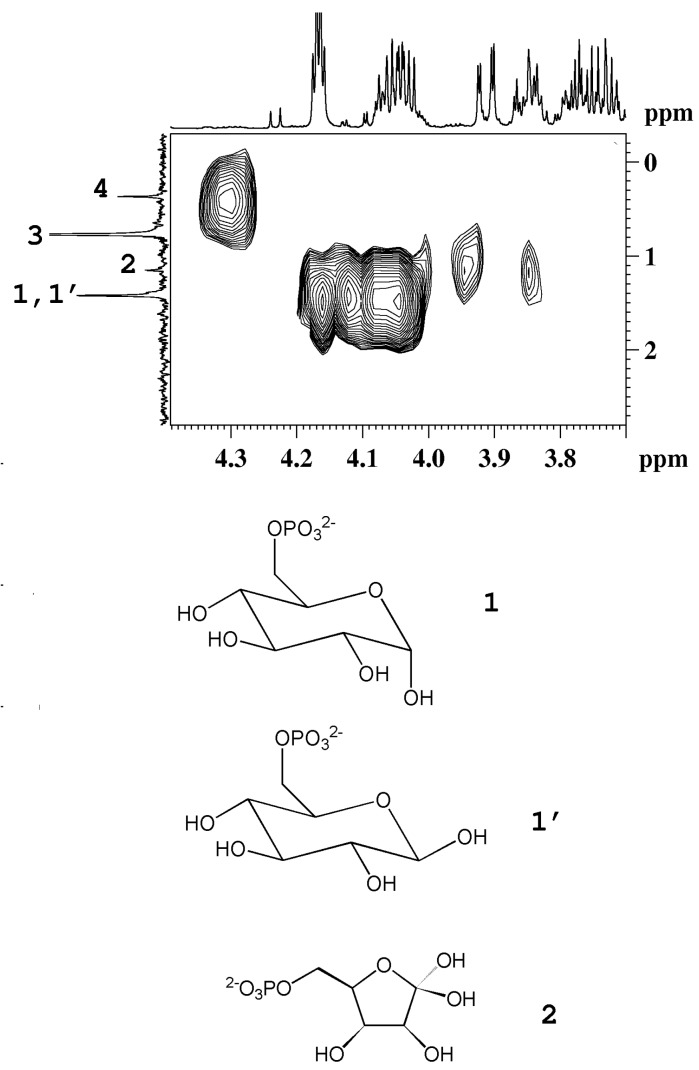
^1^H-^31^P HMBC map of an aqueous extract of kiwifruit. The ^1^H and the ^31^P{^1^H}-decoupled NMR spectra are reported as projections in the F2 and F1 dimensions, respectively. 1, 1', α-glucose-6-phosphate and β-glucose-6-phosphate; 2, fructose-6-phosphate; 3, orthophosphate; 4, unassigned compound (adapted from [6]).

Sugars such as glucose, sucrose and fructose have been detected in all fruits, whereas others, present in minor concentration, have only been detected in some fruits. For instance, trehalose was detected only in mandarin orange [16] and melon fruits [21], whereas raffinose is seen only in kiwifruits [6]. Sugars can be identified easily by means of chemical shifts and coupling constants of their anomeric protons. Usually the signals of non-anomeric protons are not useful for identification, being overlapped in a narrow region between 3.2 and 4.0 ppm. However, in some specific cases, the non-anomeric proton signals are out of this range and therefore can be used for sugar identification. It is the case of the doublet at 1.28 ppm ascribed to CH_3_ group of β-rhamnose detected in peach and mango. The detection of phosphorylated sugars (fructose and glucose) in peach and kiwifruits has been achieved by means of ^31^P NMR experiments, see Figure 4.

Regarding amino acids, alanine and valine are almost ubiquitous, whereas glycine and methionine have been detected only in black raspberry and grape, respectively. Ornithine has been detected only in orange and mango, whereas lysine has been detected in kiwifruits and mango. Pyroglutamate has been observed only in melon. 

Apart from ubiquitous metabolites, fruits contain specific secondary metabolites, usually present in minor concentrations compared with primary metabolites. These metabolites are often important from the nutraceutical point of view having antioxidant, anti-inflammatory and antimicrobial activities. Some of the secondary metabolites are characteristic for specific fruits and thus can be considered “markers” of the product. 

For instance, NMR analysis has enabled the detection of phloretin and its glucoside conjugate phloridzin dihydrochalcones, specific antioxidants in apples [14]. Another secondary metabolite is narirutin, which is specific for *Citrus* genus fruits and has been identified in orange juice [13]. According to NMR analysis [16], mandarin orange fruits contain synephrine, a bioactive compound unique to *Citrus* [25] with vasoconstrictor and bronchiectatic properties [26] and effects on weight loss/weight management [27].

Some secondary metabolites are frequently observed in different fruits. For instance, trigonelline, an alkaloid with health-promoting properties (hypoglycaemic, hypocholesterolemic, antimigraine activities) [28] has been detected in NMR spectra of grapes, mandarin orange, melon, and peach.

The observation of secondary metabolites in the ^1^H-NMR spectra of crude extracts can be hindered by the presence of major components. In fact, the difference between the concentration of the major and minor components of a metabolite mixture can be beyond the limited dynamic range of a NMR spectrometer. In this case, the limit of detection for the minor component in the mixture is higher than the limit of detection for the same compound isolated from the mixture. Therefore, sometimes, in order to obtain more detailed information on specific minor compounds, a targeted NMR approach can be complementary to untargeted NMR as in the case of blueberries, where targeted NMR analysis focused on phenols and untargeted NMR metabolite profiling have been carried out [23]. Phenolic acids and anthocyanins have been isolated using solid phase extraction (SPE) with C18 column, afterwards the mixture of CD_3_OD and CF_3_COOD (95:5 v/v) has been used as a solvent to obtain high resolution NMR spectra. Five anthocyanins have been identified, namely, malvidin-3-glucoside, malvidin-3-galactoside, delphinidin-3-glucoside, delphinidin-3-galactoside, and petunidin-3-glucoside. Interestingly, only traces of cyanidin glycosides have been detected, despite the fact that usually cyanidins are the major components of anthocyanin fraction in blueberry. Besides anthocyanins, other phenolic compounds such as 3-*O*-α-l-rhamnopyranosyl quercetin and chlorogenic acid have been identified by NMR.

### 2.2. Quantitative Analysis and Statistical Elaboration

The application of NMR in fruit metabolite study is not limited to qualitative analysis. NMR is also a robust quantitative technique provided that the following NMR experimental conditions are satisfied. The NMR signal area is proportional to the metabolite concentration provided that the complete relaxation of metabolite nuclei is achieved, therefore a recycle time (the time between successive spectral scans) of at least five times the longitudinal relaxation time (T_1_) of the slowest relaxing nuclei is necessary after a 90° pulse. The other important condition, a suitable signal-to-noise ratio, must be satisfied by choosing a proper number of scans. The NMR sensitivity is considered one of the main limitations in its application to metabolomic analysis. However, continuous developments in hardware as well as the invention and development of cryogenic probes [29] have allowed the sensitivity of NMR to be much increased.

The integration of a specific signal area *vs.* the signal area of reference compound can be used for absolute quantification yielding the amount of a specific metabolite in a given sample. Apart from the integration, other methods can be used to extract quantitative data from NMR spectra.

An NMR spectrum itself represents a metabolic “fingerprint” of the sample under investigation and can be used as such, without identification of selected signals. Instead of using the integral of selected resonances, the whole spectrum can be divided into relatively narrow regions, typically 0.02–0.06 ppm each one, and the total area within each region is calculated and considered in further analysis. This frequently used type of treatment, called bucketing or binning, has the advantage to overcome the problem of peak position and line width changes across the samples.

When a large amount of data is generated and the extraction of the desired information becomes complicated, chemometric methods are applied. Explorative unsupervised and supervised classification methods used in fruit NMR analysis are widely described in many papers and reviews [10,30,31]. 

The number of metabolites detectable simultaneously by NMR depends not only on the sensitivity and dynamic range but also on the selectivity of the extraction and on the number of extractions. In addition, some plant metabolites are bound in the cell, and are hard to extract. Therefore, the number of compounds actually present in a fruits extract after just one extraction is much smaller than the number of metabolites really present in the fruit. Only a small part of metabolome, “the tip of the iceberg”, can be detected in a single NMR experiment even by employing high magnetic fields and cryoprobes. However, although the number of compounds detected in an extract is limited, the NMR spectra give a good picture of what actually occurs in the sample. Minor compounds might not be revealed, but the major trends are clear.

The quantitative elaboration of the metabolic profile along with statistical analysis can be effectively applied for assessing some important physiological and botanical aspects of fruits such as development and ripening, variety discrimination, quality control and geographical origin. Examples of these applications are given below. 

#### 2.2.1. Development and Ripening 

NMR can be used to monitor the changes in metabolic profile of fruits during development and ripening and to provide objective criteria for determining the proper stage of fruit maturation. Moreover, it is possible to monitor metabolite composition of different fruit varieties useful to industrial applications.

Biochemical changes of black raspberry fruits at different stages of maturation (from unripe green to fully ripe dark red fruits) have been monitored by NMR coupled with multivariate statistical data analysis [19]. Two extraction solvents (CH_3_OH/H_2_O 1:1, or ethyl acetate) have been used and the metabolites extracted by CH_3_OH/H_2_O were solubilized in D_2_O or in a mixture of D_2_O with CD_3_OD and trifluoroacetic acid. Using different NMR solvents for dissolution of the same extract, noticeably different NMR spectra were observed. In fact, although totally 36 metabolites were identified, only 18 of them were clearly observed in both solutions. Only two metabolites have been assigned in the ^1^H spectra of ethyl acetate extracts leaving aside the assignment of the major part of the spectrum. Binning of selected regions in ^1^H-NMR spectra was applied for quantitative analysis and PCA and PLS-DA chemometric methods. According to NMR data, sucrose and most of the amino acids and organic acids decreased, whereas fructose, glucose, and cyanidins increased in relative concentration during maturation of black raspberry fruits. The levels of the metabolites identified in the ^1^H-NMR spectra show substantial changes during fruit ripening and the application of PLS-DA allowed the samples to be grouped according to the stage of ripeness.

The results reported by Gil *et al.* [18] described the use of high resolution magic angle spinning (HR-MAS) and liquid-state NMR spectroscopy to follow the ripening induced postharvest compositional changes in intact mango pulp and in mango juice, respectively. About 40 metabolites other than the main sugars have been identified in mango samples at different ripening stages. In pulp sucrose predominated over fructose and glucose at most ripening stages. The variation of each sugar with ripening in pulp has been in agreement with that reported previously for mango juice. On the other hand, in the juice the relative sugar composition and its variation with ripening differed significantly from that reported in literature. A possible explanation is that sucrose hydrolysis occurred to some extent before recording juice spectra. Citric acid has been the most abundant organic acid in unripe mango, however it decreased abruptly after the initial stages of ripening. During ripening a significant increase in alanine and a lesser increase in lactate, GABA, phenylalanine, and niacin, have been detected. It should be emphasized that amino acids, such as alanine, valine, leucine, isoleucine, phenylalanine and aspartic acid, are involved in aroma biosynthesis in fruits (2- and 3-methyl butanal, 2- and 3-methyl butanol, phenylacetaldehyde, 2-phenylethanol, methyl salicylate). Changes observed in the aromatic spectral region of the pulp indicate the complex chemistry of polymeric phenolic compounds and suggested the possible role of formic, gallic, and shikimic acids, and tyrosine as precursors in the polymerization processes. 

**Figure 5:**
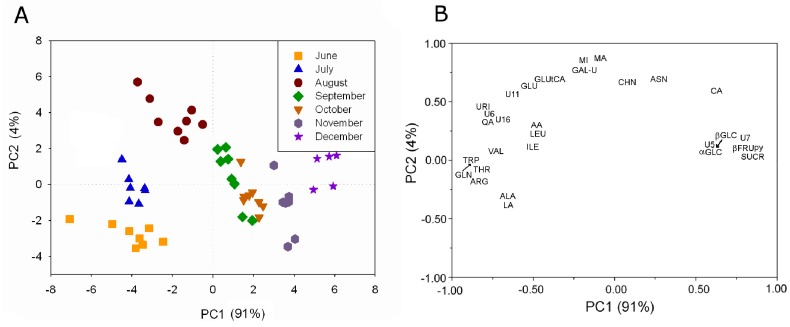
PLS analysis applied to the intensity of 29 ^1^H resonances in kiwifruit aqueous extracts always present during the investigated period: (**A**) plot of PC sample scores and (**B**) plot of variable loadings (adapted from [6]).

The metabolite profiling of aqueous extracts of kiwifruit (*Actinidia deliciosa* (A.Chev.) C.F.Liang & A.R.Ferguson, Hayward cultivar) grown in Italy has been monitored over the production season from June to December using ^1^H high resolution NMR [6,17]. About 40 water-soluble metabolites have been assigned by means of 1D- and 2D-NMR experiments. The metabolite profiling has been used to investigate the kiwifruit development during the season. The analysis has shown that some metabolites were always present, although in different concentration, whereas other metabolites were present only in some months. The intensity of 29 ^1^H resonances always present from June to December has been used in PLS analysis, see Figure 5A. 

A clear grouping of kiwifruit samples according to the harvesting month is observable. Samples harvested in June, July, and August are grouped according to the specific campaign mainly along PC2. Samples collected later in the season are separated according to the specific campaign mainly along PC1. The effect of the growth is visible as a trend along PC1, in fact the centroid of each campaign group moves toward high PC1 values according to the kiwifruit growth. The contribution of the variables to this trend given by the variable loading shows that sucrose, fructopyranose, glucose, and citric acid were present in the highest concentration in the last months. Many amino acids such as valine, isoleucine, threonine, triptophane, leucine, alanine, arginine and glutamine, and some organic acids such as lactic acid, ascorbic acid, and quinic acid were present in the highest concentration in the first period.

The metabolites such as shikimic acid, O^3^-β-d-glucopyranosyl-*trans*-caffeic acid, O^3^-β-d-glucopyranosyl-*cis*-caffeic acid, epicatechin, and 3-*O*-α-l-rhamnopyranosyl quercetin present only in the first three months are intermediates of processes that can be observed only in this period. For example, shikimic acid, a biosynthetic precursor of phenylalanine, tyrosine, tryptophan, tannins, flavonoids and lignans, has been detectable only in June and July indicating its increased utilization over this time range in the production of some volatile compounds (*p*-cresol, vanillin) and metabolites involved in the phenylpropanoids pathway, such as O^3^-β-d-glucopyranosyl-*trans*-caffeic acid. On the other hand, the observed increase in the O^3^-β-d-glucopyranosyl-*trans*-caffeic acid level occurring in July and August might be associated also to the decrease in tryptophane level.

#### 2.2.2. Varieties Discrimination 

The metabolic profile of fruit varieties can give indications regarding sensorial properties and can be also useful to face some agronomic problems. For instance, in the case of peaches, fruit fly *C. capitata* attack results in a peach disease, causing economically important losses. One of the most promising approaches to limit the chance of attack is the development of varieties naturally resistant to insect attacks. Through the cross-breeding of varieties resistant to attacks with ones possessing good agronomic qualities, it might be possible to obtain a balance between those properties. Therefore, it is important to determine the molecules and molecular mechanisms involved in defence against insect attacks. In the paper by Capitani *et al.* [24], the metabolic profiling of peaches from two varieties, Flaminia (FP) and Percoca Romagnola 7 (PR7P), has been reported. FP, a Fayette-Fairtime cross-breeding variety is characterized by good sensorial properties and is used for fresh market but is easily attacked by *C. capitata*. On the other hand, PR7P, which is a clone identified within a Percoca population mainly commercialized in a local market and used also for the canning industry, is relevant in the selective breeding to increase the resistance to insect attacks. The NMR-based metabolic profiling has shown that the pulp of PR7P has contained greater amounts of alanine, quinic acid, chlorogenic and neochlorogenic acids than FP. These molecules have been reported to be related to the defence against fungal and insect attacks in other members of the Rosaceae family [32], suggesting that the phenylpropanoids pathway was at least partially involved in the repulsion of *Ceratitis capitata*. For instance, the metabolism of alanine and quinic acid leads to the biosynthesis of volatile compounds such as benzaldehyde and benzyl alcohol characterized by the ability to repel herbivore attacks [33]. At the same time, PR7P variety is poorer in valine and isoleucine [34] correlated with the formation of branched medium-length chain alcohols, acids, and aldehydes, which are volatiles known to attract the insect.

The investigation of metabolite composition of different fruit varieties can also give an important contribution to the development of data bases for the detection of adulterations and frauds of fruit juices. Many factors, such as geographical origin, storage, fruit varieties, and processing can potentially influence the metabolic profile of juices and must be taken into account. Preliminary studies on apple juices from different cultivars, years of production, and duration of storage have shown that the cultivar has been one of the most important sources of metabolite profile variability [14,35,36]. The levels of malic acid, phenolic compounds (epicatechin, phloridzin-phloretin, *p*-coumaric and chlorogenic acids) and sugars (glucose, fructose and sucrose) have been reported to be dependent on cultivar and hence mainly contributing in the differentiation of metabolite profiles of juices from different apple cultivars. According to a recent study [15], juices from the same cultivar but different geographical origin can have a different metabolite profile. ^1^H-NMR spectra of apple juices and pulp aqueous extracts from five different cultivars and two different regions (Japan and New Zealand) have been compared. Samples of Fuji cultivar from Japan contained larger amount of l-rhamnitol than New Zealand apples of the same cultivar. Moreover, sugars (sucrose, glucose, xylose), and acids (citramalic and quinic acids) have shown significant differences as well. 

Another important aspect to take into account is the metabolic change during the process of juice production. Studies performed directly, without extraction, on fresh apple tissues using high resolution magic angle spinning (HR-MAS) ^1^H-NMR spectroscopy have shown distinct differences in some metabolites between apple tissue and juice [37]. For example, ethanol and acetaldehyde present in pulp were absent in juice, whereas lactic acid appeared in juice only. Apart from these differences between pulp and juice, the HR-MAS ^1^H-NMR study of metabolite profile of apple tissues derived from 3 different cultivars confirmed the influence of cultivar on the metabolite composition. The levels of malic acid, alanine, acetaldehyde, chlorogenic acid, epicatechin, sucrose and glucose were found to be different in the three cultivars. 

Kiwifruits that include various *Actinidia* species have become an important horticultural crop for their sensory and nutritional properties. For many years only one fruiting cultivar, “Hayward”, has dominated the international market. Recently, commercial cultivation of different *Actinidia* species with quite distinct fruits has begun to take hold. For example, a kiwifruit cultivar “CI.GI” crossbreed from different species of *Actinidia deliciosa* (A.Chev.) C.F.Liang & A.R. Ferguson has been developed to obtain kiwifruits characterized by an earlier ripening than that of Hayward kiwifruits. In this way, it would be possible to have mature fruits even when Hayward kiwifruits are still unripe. Among other *Actinidia* species, Hort16A kiwifruit from *Actinidia chinensis* Planch, marketed under the name of Zespri Gold has assumed a certain commercial relevance being characterized by a yellow flesh and a flavor sweeter than that of Hayward kiwifruit. ^1^H-NMR has been applied to characterize the three kiwifruit cultivars mentioned above by assessing their metabolite profiles [17]. 

The metabolite profiling of Zespri, CI.GI, and Hayward kiwifruits displayed common metabolic features, such as high levels of quinic, citric, and ascorbic acid, but also important differences in the metabolic behavior during the growth and development of fruits [17]. For instance, the amount of O^3^-β-d-glucopyranosyl-*trans*-caffeic acid was found to be higher in immature Hayward and CI.GI kiwifruits than in Zespri. Moreover, Hayward and CI.GI immature kiwifruits showed the presence of 3-*O*-α-l-rhamnopyranosyl quercetin that was absent in Zespri cultivar. Neochlorogenic acid found only in Zespri kiwifruits can be considered a specific marker of this variety. In Hayward and CI.GI kiwifruits a significant amount of epicatechin was detected in August, whereas in Zespri the amount of this compound was found to be low over the season.

NMR data for the metabolites common to all cultivars at each developmental stage have been subjected to PCA. Metabolic profiles of Zespri kiwifruits were always separated along the first principal component axis from the other two cultivars at each stage of development, whereas PCA analysis was not able to proof differences between Hayward and CI.GI kiwifruits over the season. To evidence a possible difference between these two cultivars, PLS2 analysis has been carried out using the same variables used for PCA. The most relevant metabolites responsible for this separation were found to be arginine, choline, *myo*-inositol, quinic acid, sucrose, uridine and histidine.

The combination of ^1^H-NMR and gas chromatography-electrospray ionization time-of-flight mass spectrometry (GC-EI-TOFMS) was used for metabolomic profiling of three cultivars (Cézanne, Escrito, and Hugo) of melon (*Cucumis melo* L.) fruit [21]. The metabolite composition of melon juice and fruit flesh extracts has been evaluated, 27 and about 40 metabolites were identified and quantified by NMR and GC-EI-TOFMS, respectively. PCA analysis applied to NMR data of fruit flesh extracts has revealed cultivar specific differences in metabolite content and has shown that the hexoses (e.g., glucose and fructose) with citric acid were more abundant in Cézanne cultivar, whereas almost all amino acids and sucrose had a higher level in Escrito cultivar. Glutamic acid has been the marker for the third cultivar, Hugo. Additionally, the spatial localization of the major polar metabolites in the different slices of melon flesh (epicarp, outer mesocarp and inner mesocarp) of the three cultivars has been assessed through ^1^H-NMR and GC-EI-TOFMS profiling. The cultivar-specific gradient of metabolite concentration from the epicarp to the inner mesocarp was observed for several metabolites, for example GABA gradient in Cézanne cv, acetic acid in Escrito cv, and malic and aspartic acids in Hugo cv. A multiblock hierarchical PCA method has been applied for correlation of data from ^1^H-NMR and GC-EI-TOFMS metabolomic platforms.

The sensory quality evaluation of seven cultivars of watermelon (*Citrullus lanatus* (Thunb.) Matsum. & Nakai) grafted on two different rootstocks has been carried out and correlated with ^1^H-NMR metabolic profiles [22]. Twelve major metabolites have been assigned in the ^1^H-NMR spectra of juice extracted from the flesh tissues. Quantitative NMR data have been obtained using binning of entire spectrum except for water resonance, and PLS-DA have been employed as the supervised pattern-recognition method. The results have demonstrated that the quantity of sugar metabolites in the central flesh tissues was largely dependent on the rootstock cultivar, whereas, the watermelon cultivar had no significant effect on the sugar quantity and therefore on sensory properties of the flesh tissues from the central part of the watermelon. Based on the sensory evaluation, it could be inferred that sucrose was the predominant biochemical component contributing to the sweetness of watermelon.

#### 2.2.3. Quality Control 

Fruit juice profiling by ^1^H-NMR has been suggested as a method for authentication and verification in the quality control of fruit juices [38]. This approach has allowed the quantification of more than 20 different compounds together with a fully automated screening using statistical models for the estimation of fruit content or the type and origin of the juice.

After having established a spectral database containing spectra of more than 3000 reference juices of about 1000 fully authentic samples in 2009, a constant update of the reference juice database is maintained. A specific case of adulteration, namely the addition of pulp wash to orange juice, has been evaluated using NMR metabolomics approach by Le Gall *et al.* [13]. Dimethylproline has been identified as a potential marker for the detection of pulp wash addition using PCA as unsupervised statistical analysis. 

The detection of other frauds such as sugar addition, exhaustive enzymatic treatment, addition of citric acid or lemon juice, extraction of orange peel, or use of unripe fruits has been possible using fruit juice profiling by NMR [39]. Marker compounds like sucrose, galacturonic acid, and phlorin have been identified and quantified. To detect anomalies in the origin of a sample, the fruit content or the addition of other types of fruit, a non-targeted approach together with statistical methods has been applied. A large reference database has been developed with more than 6000 samples of more than 50 different types of fruit juices from more than 50 countries. 

#### 2.2.4. Geographical Origin 

The effects of elevation, rootstock, and soil depth on the metabolic profile of mandarin orange juices prepared from fruits harvested in 11 different locations in California have been investigated by NMR [16]. About 30 metabolites have been identified, quantified and processed by multivariate statistical analysis.

In general, trees grown at a higher elevation tended to have higher concentrations of amino acids (specifically asparagine, threonine, phenylalanine, valine, alanine, and arginine), succinate, and 4-aminobutyrate but lower concentrations of sugars (glucose, fructose, sucrose), and limonin glucoside. Mandarin oranges taken from trees grown on trifoliate rootstock have the highest content of 4-aminobutyrate, ethanol, phenylalanine, isoleucine, and succinate. On a grove-to-grove basis, a 5-fold or even higher differences between the highest and the lowest concentrations of arginine, asparagine and proline have been observed. Some components, such as amino acids, limonin glucoside, 4-aminobutyrate, synephrine, trigonelline, and proline betaine, ranged from under 1 mM to several millimolar. Sugars, acids, and aromatic compounds are considered the major components contributing to fruit quality and the fact that metabolite concentrations can vary so greatly depending upon the growth conditions of a grove suggests that fruits from each grove may have a particular taste profile.
